# Characterizing iconic gesture during narratives in chronic traumatic brain injury recovery

**DOI:** 10.3389/fnhum.2024.1393284

**Published:** 2024-11-25

**Authors:** Katelyn Urena, Brielle C. Stark

**Affiliations:** ^1^Department of Speech, Language and Hearing Sciences, Indiana University Bloomington, Bloomington, IN, United States; ^2^Program in Neuroscience, Indiana University Bloomington, Bloomington, IN, United States

**Keywords:** “traumatic brain injury”, gesture, narrative, longitudinal, communication, language, “iconic gesture”

## Abstract

**Introduction:**

It is known that co-speech hand gestures increase and supplement speech in individuals with language impairment after brain injury, e.g., post-stroke aphasia. Traumatic Brain Injury (TBI) provides a unique avenue to evaluate gestures as TBI often presents with both anomia (word-finding impairments) and cognitive impairments, resulting in a cognitive-communicative disorder. However, there is a great need for evaluation of gestures in TBI during typical spontaneous speech and across the recovery trajectory (from sub-acute to chronic stages). In a large population (*N* = 54) of persons with moderate-severe TBI, who were examined at 3 months post-TBI whilst telling a procedural narrative (“how to make a sandwich”), we examined three aims: (1) characterize the extent to which adults with moderate-severe TBI produce iconic gestures; (2) identify the extent to which language impairment relates to iconic gesturing in TBI; and (3) characterize the extent to which iconic gesturing changes across TBI recovery.

**Methods:**

In a subpopulation (Group 1, *N* = 14) who were examined at three- and 24-months (sub-acute and substantially chronic), and in a smaller subpopulation (Group 2, *N* = 6) who had data for five timepoints (three-, six-, nine-, 12-, and 24-months), we used paired tests to examine and characterize longitudinal changes in iconic gesturing.

**Results:**

The large group analysis suggested that individuals with TBI use iconic gesture during narrative, which take several different iconic forms (e.g., enacting use of an object), and that a minority employed gestures that supplemented (added to, disambiguated, or replaced) speech. The subpopulation analyses suggested that participants did not produce iconic gestures significantly differently across the 2-year recovery timeframe. Case examination of a participant with moderate-severe aphasia suggested a relationship between language impairment and gesture, with this individual producing the highest proportion of supplemental gesturing of the entire group. This finding aligns with research from the post-stroke aphasia field.

**Discussion:**

Broadly, this study significantly extends prior research on the relationship between gesturing, language, and brain injury.

## Introduction

### Gesturing during speech

**FIGURE 1:**
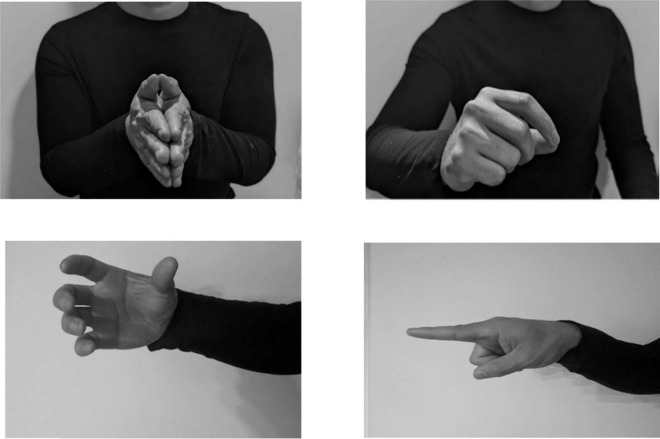
Iconic gestures, which are subcategorized into iconicity representation types, from top left to bottom right: enacting, handling, object, and path (if moved from one place to another).

Co-speech hand gestures are naturally occurring, ubiquitous, and thought to aid in cognitive processes, like language, memory, and learning (Goldin-Meadow, 1999, 2017; Kendon, 1994; Kita, 2009; Kita et al., 2017; McNeill, 1992). Representational gestures are a group of gestures that are connected to speech content and therefore carry meaning (McNeill, 1992). The representational subgroup includes iconic, metaphoric, and deictic gestures. Iconic gestures have a close relationship to the semantic content of speech (Figure 1), metaphoric gestures present abstract ideas (e.g. pretends to grab an object “I have to grab those sales”), and deictic gestures involve pointing to denote concrete events and objects, and to refer to abstract referential spaces (e.g. index finger pointing to the left “when you enter my house, the kitchen is on the left”). According to van Nispen et al. (2016), iconic gestures can be further subclassified into iconicity types, which provide more information about the intent of the gesture. These types include *enacting* (pretending to perform an action), *handling* (pretending to use a tool), *object* (some part of the hand represents part of an object or is holding an object), *shape* (outlining the shape of an object), and *path* (outlining the path from one space to another). See Figure 1 for an example of four iconicity types, which are all iconic gestures.

Representational gestures whose function is to supplement (i.e., add to, disambiguate, or replace) speech have been identified repeatedly across age groups and serve different communicative functions. An example of a supplemental, adding gesture would be to show the size of a ball using the hands whilst saying “ball.” In this case, the gesture adds a dimension (size) that speech does not. Children ages 14–34 months have been shown to rely on gestures to compensate for their limited ability to articulate spoken language (Özçalışkan and Goldin-Meadow, 2009), indicating early markers for language development (e.g. “eat” + point to fruit). Children between ages four and five have been shown to use supplemental gestures when describing causal relationships between objects and events, suggesting that older children use gestures to support complex ideas that cannot be expressed through speech (Göksun et al., 2010). In cognitively healthy adults, the use of supplemental gestures is less common, indicating a shift in the function of gestures during development (Alibali et al., 2009). That is, adults tend to use gestures redundantly with speech, to emphasize information or align with what is being spoken, rather than supplement speech. This was confirmed by a recent study, where cognitively healthy older adults produced gestures that primarily matched information already stated in speech (Özer et al., 2019).

This introduction outlines gesture production across the lifespan and after brain injury, presenting a need to evaluate gesture and its function in a novel population (traumatic brain injury).

### Gesturing across the lifespan

The use of gesture in children is an extensively studied topic, with gestures found to aid in cognitive processes such as learning (Goldin-Meadow, 2009; Goldin-Meadow et al., 2001), memory recall (Cameron and Xu, 2011; Church et al., 2007; Stevanoni and Salmon, 2005), and language (Rowe and Goldin-Meadow, 2009). For example, one study found that children learn math better when their teacher gestures versus when they do not (Singer and Goldin-Meadow, 2005). From infancy and into childhood, gesture has shown phenomenal cognitive benefits and holds an essential role in communication that has led to further questions about the role of gesture during development and in older adulthood.

Previous studies have demonstrated that children and adults exhibit differences in gesture production. Colletta et al. (2010) examined age-related changes in co-speech gesture production and found that adults (aged 18–33) produced shorter narratives with increased complexity and gesture frequency than children. Additionally, they found an increase in gesture frequency in 10-year-olds compared to 6- year-olds after completing the same narrative task. These results suggest that gesture use becomes more frequent and complex over time. In a recent study involving both younger and older adults, participants were tasked with describing concrete and abstract images. The results revealed that, irrespective of the context, older adults displayed fewer representational gestures but produced more deictic gestures than young adults (Arslan and Göksun, 2022). Another study focusing on eliciting visual imagery, motor imagery, and abstract concepts observed that older adults exhibited fewer representational gestures specifically during the visual imagery condition, yet gesture rate remained comparable between younger and older adults for the other conditions (Feyereisen and Havard, 1999). When perceiving speech accompanied by gesture, younger adults appear to be more able to integrate gesture into their knowledge/comprehension than older adults when speech and gesture are integrated simultaneously (Cocks et al., 2011). These studies suggest that gesture production and comprehension differ between younger and older adults, and that this depends upon the task and complexity of the condition. In sum, the literature makes a compelling case for the fact that gestures occur often during natural speech in both younger and older adults, with some differences in how older adults use and comprehend gestures, though this is a continuing area of study.

### Gesture use after brain injury

There is a growing body of knowledge about how gesture becomes particularly important for enhancing communication when language is impaired after brain injury, with the bulk of this research occurring in post-stroke aphasia. Ischemic strokes represent the third major cause of morbidity and mortality worldwide, with left hemisphere strokes, especially those affecting the middle cerebral artery territory, resulting in chronic aphasia (Simmons-Mackie and Cherney, 2018). Aphasia can impact language production and comprehension skills. Persons with post-stroke aphasia often elicit more iconic gestures than cognitively healthy peers when spontaneously speaking (e.g., Kong et al., 2017). Notably, the use of gestures to *supplement* speech also increases in the presence of acquired language impairment. Individuals with post-stroke aphasia have been noted to employ a higher rate of supplemental gestures compared to cognitively healthy peers (Cocks et al., 2013; Hogrefe et al., 2013; Kong et al., 2015, 2017; Stark and Oeding, 2023; van Nispen et al., 2017), and with individuals who have a greater language production impairment tending to produce a higher rate of supplemental gestures (Sekine et al., 2013; Stark and Oeding, 2023). These findings suggest that gesture may be able to support communication when language production is impaired (as in aphasia), supporting theory that gestures complement language, yet arise from a different interface (i.e., action imagery system) (Hostetter and Alibali, 2019; Kita et al., 2017). That gesture and language share some basic processes (e.g., access to semantics/meaning), but largely diverge, is one explanation for why gesture is often employed by individuals with aphasia despite severe language impairments. Note that this statement is controversial: some individuals with severe Wernicke’s aphasia, or semantic impairment, may not use gestures that carry meaning (Cicone et al., 1979), though a recent review suggests that persons with severely impaired semantic access can use gesture communicatively (de Kleine et al., 2024).

Although it is well understood that gestures occur more frequently in individuals with post-stroke aphasia, and that gestures often supplement speech, few studies have investigated co-speech gestures across different brain injury etiologies (Clough et al., 2023; Clough and Duff, 2020). Traumatic brain injury (TBI) is caused by an external force to the head, and because of its diffuse effects on the brain, often leads to cognitive impairment, specifically impairments of memory, and in some cases, can result in aphasia (Heilman et al., 1971). In a longitudinal study of adults with TBI, cognitive fatigue and word finding difficulties were most common acutely and did not improve after 10 years post injury (Ponsford et al., 2014). As such, people with TBI may present with similar symptoms to post-stroke aphasia such as word retrieval difficulties and exhibit in similar behaviors such as circumlocution, lengthy pauses, and difficulty with language production (Hough, 2008). In a study on communication after severe TBI, it was noted that TBI may impair an individual’s use of pragmatics and non-verbal communication, including manual and facial gestures (Rousseaux et al., 2010). However, the study employed a standard assessment (Lille Communication Test) rather than evaluating the extent to which gestures were employed spontaneously in everyday conversation. In another study, it was found that individuals with TBI utilize general gestures more than their healthy peers whilst naming pictures (Kim et al., 2015). The two studies on gesture usage in TBI, discussed above, evaluated gesture in isolated instances (e.g., on a standard test; on a naming battery), which does not indicate the extent to which individuals with TBI employ gesture in more natural communication settings, such as storytelling. Interestingly, recent evidence suggests that individuals with TBI do produce iconic gestures during spontaneous speech tasks. For instance, (Clough et al., 2023) found that individuals with TBI were just as likely as their non-injured peers to produce iconic gestures when retelling stories. However, there is still limited understanding of the communicative functions these gestures serve or the types of information they convey.

Moreover, changes in gesture use during the recovery process (i.e., months after the injury, when many brain changes are still occurring) and the extent to which gesture is used to supplement speech requires further investigation. For example, it remains unclear whether individuals with TBI who are experiencing more severe language impairments use gestures to supplement speech more often than individuals with TBI who present with milder language impairments. Evidence from aphasia drives the hypothesis that individuals with TBI and more severe language impairments would indeed use gestures in a supplemental fashion more often than those with milder language impairments.

There is a great deal of benefit for evaluating gesture during typical communication scenarios in individuals with TBI. To evaluate the relationship between gesture and language (when language is and is not impaired) has generated evidence to support the variety of gesture theories in the literature (de Beer et al., 2019, 2020; de Ruiter, 2000; Hostetter and Alibali, 2019; Kendon, 2011; Kita et al., 2017; Krauss, 1998; McNeill and Duncan, 2011). McNeil’s growth point theory suggests that both speech and gestures constitute a single system, stemming from a unified conceptual source, thus co-speech gestures should be interpreted as a single unit (McNeill, 2000, p. 20). This theory, however, does not explain how gestures and speech are able to precede lexical items, or how gestures may aid in lexical retrieval. Levelt (1992) model of speech production describes the cognitive and linguistic processes from converting concepts to spoken language. This process includes conceptualization, formulation, and articulation of a message, and has been utilized frequently to develop frameworks for speech and gesture integration. Expanding Levelt’s model, the Lexical Retrieval Hypothesis (LRH) emerges from the observation that gestures often precede words and posits that gestures stem from the level of speech (though unclear if semantic or phonological), thus aiding in retrieval of lexical spatial items (Krauss and Hadar, 1999). The Sketch Model proposes that gestures stem from a distinct source: gestures are utilized to convey concepts with spatial and motoric properties, with a system (conceptualizer) responsible for planning production of gestures (de Ruiter, 2000). The Sketch Model was expanded to show how speech shapes gesture via an action and message generator (gesture-for-action hypothesis), which communicate bidirectionally (Kita and Özyürek, 2003). A recent study investigated whether preventing individuals from gesturing would increase the rate of speech disfluencies, which would provide evidence for the LRH. This study found no differences in speech disfluencies for any categories of speech (i.e., literal or metaphorical spatial content), indicating that gestures did not help locate words for spatial contents (Kısa et al., 2022). Thus, although various theories exist regarding the integration of gestures and speech and it is clear that there is a connection between gestures and speech, the extent to which gestures are employed and are organized cognitively remains an ongoing area for investigation.

Investigating gesture use after a TBI may reveal how cognitive resources are distributed. In a study on verbal working memory and gesture production, it was noted that lower verbal working memory was associated with higher likelihood of gesturing. That is, an individual’s verbal working memory capacity is linked to gesturing, thus supporting that gesture lightens the load for younger adults without brain injury (Gillespie et al., 2014). In the case of TBI, it has been numerously reported that individuals with TBI have lower verbal working memory capacity, as they show poorer performance on executive working memory tasks compared to their healthy peers (Vallat-Azouvi et al., 2007). Moreover, it has also been noted that individual differences in cognitive skills are associated with gesture production (Özer and Göksun, 2020). A study on verbal and spatial skills found that phonemic fluency skills (i.e., organizational efficiency) were associated with representational gesture use in younger adults. Additionally, individuals with both lower phonemic fluency and higher spatial skills had higher gesture rates (Hostetter and Alibali, 2007). These studies demonstrate that individual working memory skills and individual cognitive skills both contribute to gesture production. While it is well documented that TBI impacts executive functioning, social communication, and other cognitive skills, it is still unclear how cognitive changes extend to gesture use. Investigating the use of natural use of gestures in TBI may reveal compensatory strategies and adaptation of communication modalities during communication recovery.

There are also clinical implications for understanding gesture use post-TBI. For example, investigating gesture use post TBI may reveal the extent to which gestures are used by those with brain injury where language impairment is not as severe (or present), and how gesture use changes across their recovery period. If indeed individuals with TBI have a tendency to use gestures immediately after their injury in a communicative way−for example, using iconic gestures, and/or gestures that supplement speech in some way [via added information or even pragmatically]−this would suggest that a clinician, like a speech-language pathologist, should assess and leverage gestures when working to improve communication abilities in this person.

### Aims

Aim 1: Characterize the extent to which adults with moderate-severe TBI produce iconic gestures. No hypothesis drove this question, given the limited research evaluating iconic gesturing in TBI during spontaneous speech settings.

Aim 2: Identify the extent to which language impairment relates to iconic gesturing in TBI. Drawing a parallel from findings in post-stroke aphasia cases, where heightened gesture usage aligns with greater language impairment, we hypothesized that participants with more severe language impairment would produce more iconic and supplemental gestures.

Aim 3: Characterize the extent to which iconic gesturing changes across TBI recovery. Due to participants scoring within normal limits on neuropsychological assessments and demonstrating fluent intelligible speech we hypothesized that in a paired longitudinal analysis, the rates and function (e.g., supplemental) of gestures will not significantly change across a two-year recovery, from sub-acute (three months) to chronic (two years).

## Methodology and design

### Participants

Archival audiovisual data were extracted from TBI TalkBank (tbi.talkbank.org), a shared database of multimedia interactions for the study of communication in people with traumatic brain injury. While this database houses audio and visual data from a variety of labs around the world, the data for the current study were extracted from a longitudinal study investigating communication recovery following moderate-severe TBI in Australia (Elbourn et al., 2019). Video data during a procedural narrative task (described in more detail, below) were extracted for N = 57 Australian English participants (n = 11 bi- or multilingual). All participants were classified by the original study as having moderate-severe TBI. The original study collected a variety of neuropsychological assessments, including cognitive and communication measures such as the Western Aphasia Battery – Revised (a commonly used standardized aphasia battery; Kertesz, 2007), Boston Naming Test (a confrontation naming assessment of nouns; (Kaplan et al., 2001), Northwestern Verb Naming Test (a confrontation naming assessment of verbs; Cho-Reyes and Thompson, 2012), and the La Trobe Communication Questionnaire-Self (a specialized self-report assessment for TBI about perceived communicativeness; Douglas et al., 2000). These neuropsychological assessments were only administered at three months post injury, while the procedural narrative was collected three-, six-, nine-, 12-, and 24-months post injury. Several individuals had mild dysarthria, and none exhibited signs of apraxia of speech. Data for limb apraxia were not collected. Notably, n = 22 participants presented with aphasia quotients less than 93.8 from the Western Aphasia Battery – Revised, which is indicative of clinical aphasia. One participant received an aphasia quotient of 55, indicating moderate-severe aphasia.

#### Exclusion of data

Because this study evaluated gestures, data from participants at each time-point was kept only if the arms and hands were clearly visible in the camera frame for the duration of the task, and if the camera lighting and clarity was acceptable for distinguishing gestures. Out of the initial dataset of 57 participants, three individuals were excluded from the study because they had no video files available at any timepoints. Participants were not excluded if they did not gesture.

After exclusion, *n* = 31 had data at three months; *n* = 31 at six months; *n* = 19 at nine months, *n* = 36 at 12 months, and *n* = 30 at 24 months. Participants that did not gesture at a given timepoint resulted in a gesture count of 0, which is documented and incorporated into the analysis. Thus, a total of 97 files were annotated for gestures for 54 individuals across all timepoints.

Table 1 summarizes the included dataset (*N* = 54).

**TABLE 1:** Demographic and neuropsychological data for included *N* = 54 adults with TBI.

Factor	M(SD) or N (%)
**Post Traumatic Amnesia Days**	
Mean (SD)	52.8 (40.4)
Median [Min, Max]	43.5 [6.00, 215]
**Sex**	
Female	10 (18.5%)
Male	44 (81.5%)
**Age**	
Mean (SD)	34 (13.3)
**Head Injury**	
Closed	52 (96.3%)
Open	1 (1.9%)
Open and Closed (multiple injuries)	1 (1.9%)
**Language**	
Monolingual	44 (81.5%)
Bilingual	7 (13%)
Multilingual	3 (5.6%)
**Western Aphasia Battery—Aphasia Quotient**	***Max Score* = *100 (higher* = *milder aphasia)***
Mean (SD)	92.5 (7.4)
Median [Min, Max]	93.6 [55, 100]
Missing	12 (22.2%)
**Boston Naming Test**	***Max Score* = *60 (higher* = *milder anomia)***
Mean (SD)	43.0 (14.2)
Median [Min, Max]	47.5 [0, 59.0]
Missing	10 (18.5%)
**Verb Naming Test**	***Max Score* = *22 (higher* = *milder anomia)***
Mean (SD)	19.8 (3.68)
Median [Min, Max]	21.0 [3.00, 22.0]
Missing	10 (18.5%)
**LaTrobe Communication Questionnaire—Self**	***Max Score = 120*** ***(higher = more perceived issues)***
Mean (SD)	47.5 (11.4)
Median [Min, Max]	45.5 [31.0, 72.0]
Missing	12 (22.2%)
**Dysarthria**	
Present	36 (66.7%)
Not Present	7 (13.0%)
Missing	11 (20.4%)
**Apraxia of Speech**	**None observed**
**Initial Glasgow Comma Scale**	***Max* = *15 (higher score* = *higher functioning)***
Mean (SD)	6.78 (3.53)
Median [Min, Max]	6.50 [3.00, 15.0]

### Discourse task

Video data were acquired for all discourse tasks from the TBIBank protocol (Togher et al., 2014), which include picture description, picture sequence exposition, fictional story retell, autobiographical narratives, and a procedural narrative (MacWhinney et al., 2011). For the purposes of the current study, the procedural narrative (“tell me how to make a sandwich”) was evaluated. The procedural narrative was selected for several reasons. Spatial language, like that which is used in procedural narratives, has been shown to correlate with a higher rate of iconic gesture use (Alibali, 2005; Kita and Lausberg, 2008). Further, prior studies in acquired communication disorder populations (e.g., aphasia) have also evaluated procedural narratives and found high rates of iconic gesture production (Pritchard et al., 2015; Stark and Cofoid, 2022). We opted to capture iconic gesturing during a task we thought would be liable to produce these types of gestures and enable characterization.

### Characterization and encoding of gesture production

#### Gesture definitions

Iconic gestures were defined using McNeill’s parameters, with reference to Sekine and Rose (2013): a gesture that depicts a concrete action, event, or object. They are reported as gesture rates per 100 words.

Gesture function was defined based on Stark and Oeding (2023). Iconic gestures were separated into two primary functions: redundant or supplemental. Only two categories were used because of notable issues in rater reliability across many function categories in other studies (Kong et al., 2015; van Nispen et al., 2017). For instance, expanding the number of categories to distinguish between gesture functions led to sparse occurrences and decreased rater reliability. Due to these findings, we chose to reduce gesture function categories into supplemental and redundant. A supplemental gesture was therefore defined as adding to, disambiguating, or replacing speech. Gesture function was characterized as supplemental if the gesture (1) conveyed information that was not uttered in the speech (e.g., gesture showed running activity whilst participant said “over there”), (2) gesture added information to the speech (e.g., shape of hands showed size of a loaf of bread when saying “you get the bread”), or if (3) only gestures were produced (no speech). An example of a supplemental gesture from this narrative task is a speaker pretending to hold a knife and spread peanut butter on bread whilst also holding a jar, during which they utter, “You put the thing on the bread.” In this case, the gesture adds information (that “thing” is something spreadable and from a jar). Moreover, here, the gesture contributes information about the manner of action, which may lack clarity in speech (such as “put”) but is informative in gesture (spread motion). An example of a redundant gesture from this narrative task is a speaker making a spreading motion whilst saying “spread it.” Supplemental gestures are always reported as a percentage of the total iconic gestures.

Iconicity types were based on studies in aphasia by Cocks et al. (2013 and van Nispen et al. (2016): enacting, handling, object, shape, path, and mixed iconicity. Gestures were encoded as enacting if the gestures had movement and if they were semantically meaningful but were not indicative of the speaker using a tool. Gestures were encoded as handling if the speaker’s intent was to pretend to use a tool. Gestures were encoded as objects if there was little movement, and if the hand represented an object or part of an object. Shape was encoded if the speaker pretended to outline the shape of the object. Path was encoded if the speaker intended to describe the path from one space to another. Gestures were characterized as mixed if two different iconicity types were utilized at the same time. Iconicity types are always reported as a percentage of the total iconicity types.

#### Gesture coding in ELAN

All gestures were annotated manually using the software ELAN (Version 6.2) in a Windows 10 environment (Sloetjes and Wittenburg, 2008). A tier was created for each hand (left hand; right hand), upon which information about the gesture function (supplemental or redundant) and type (i.e., iconic) was collected. A tier for each hand was created because some individuals used only one hand for a given gesture, while others used both hands to convey information, and sometimes, the information carried in one hand was different from the information carried in the other hand. A separate tier was created for iconicity types. A single annotation for a gesture included the preparation, stroke, hold, and retraction phases. However, gesture phases were not coded in ELAN. A new annotation is created when the hand returns to a rest position, however, the hand does not need to return to the same rest position prior to starting the gesture. Iconic, metaphoric, and deictic gestures were characterized and coded in ELAN, however, this study analyzes and reports iconic gesture rates only. See Table 2 for an example of the coding scheme. Note that within the coding scheme, RED = gestures that are redundant to speech, IC = iconic gestures, HA = iconicity type handling, and OB = iconicity type object.

**TABLE 2:** Segmentation of gestures and example of coding scheme.

Hand	Gesture description	Stroke Phase (not coded into ELAN)	Coding scheme
**Annotation 1: “You get the knife scoop the peanut butter…”**			
Right hand	Hand raises, fist closes as if holding a knife and pretends to scoop peanut butter from a jar	Preparation, stroke, and retraction	RED/IC/HA
Left Hand	Hand raises and pretends to hold a jar	Preparation, stroke, and retraction	RED/IC/OB
**Annotation 2: “then you spread it on the bread”**			
Right hand	From the retraction phase, the hand begins to make a spreading motion as if spreading peanut butter on the bread	Stroke and retraction	RED/IC/HA
Left Hand	From the retraction phase, the handshape changes into a flat open palm as if holding a piece of bread	Stroke and retraction	RED/IC/OB

**FIGURE 2:**
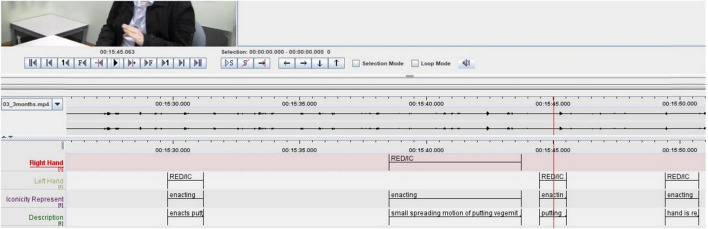
Example of ELAN interface with gesture coding tiers (left) and examples of codes (right). RED, redundant gesture; IC, iconic gesture.

Figure 2 shows an example of this study’s coding scheme in ELAN. Characterization of supplemental or redundant gestures was done by listening to the audio of the speech after all gestures were characterized. Figure 2 shows that gesture function was coded in the hand tiers, e.g., RED/IC = redundant iconic. This study does not report findings on left- or right-hand utilization. Gestures were encoded as such because the study’s aim was to examine the intention of the gestures (i.e., were the gestures meaningful, adding to speech etc.), rather than handedness.

#### Coding reliability

Ten participants were randomly selected from various timepoints to assess inter-rater reliability between two coders. For each ELAN file, the number of gestures (i.e., count of each gesture type) was recorded for each gesture type. A two-way agreement intraclass correlation coefficient was calculated to determine agreement between iconic gesture frequency (i.e., agreement of iconic gesture counts for a given individual) (Koo and Li, 2016), and Chohen’s Kappa to determine reliability of iconicity types, and gesture function. Intraclass correlation coefficient indicated excellent reliability for total iconic gesture frequency (ICC = 0.92, p = 0.014 with 95% confidence interval = 0.109–0.985). Cohens Kappa revealed moderate agreement for enacting (κ = 0.49, *p* = 0.001, z = 3.21), substantial agreement for handling (κ = 0.72, *p* < 0.001, z = 4.03), moderate agreement for object (κ = 0.44, *p* = 0.09, z = 1.69), and fair agreement for mixed (κ = 0.38, *p* = 0.24, z = 1.19) iconicity types. Cohens Kappa could not be calculated for supplemental gestures as raters reported few to no productions across all ten individuals.

### Analysis

Given the variability between samples at different time points, and the non-linear nature of the data, non-parametric statistics were employed throughout. The analysis can be conceptualized as a funnel, where Aims 1 and 2 are broad and focus on the large group (*N* = 54) and Aim 3 narrows to characterize longitudinal change in two subgroups (Group 1, *n* = 14; Group 2, *n* = 6). A case study, the narrowest point of our analysis, is also provided as evidence of a relationship between language impairment and supplemental gesturing. All analyses were completed in R version 4.1.1 and RStudio Build 576. Raw, de-identified data, and the R Markdown file, can be found in our OSF project: https://osf.io/58gqx/. Original data (video, neuropsychological and demographic data) can be found on TBIBank once you are a member (tbi.talkbank.org). Membership is free.

This study was not pre-registered.

#### Aim 1: characterize the extent to which adults with moderate-severe TBI produce iconic gestures

No hypothesis drove this question, given the limited research evaluating iconic gesturing in TBI during spontaneous speech settings. Firstly, a Kruskal-Wallis rank sum test was performed in order to assess if there were significant changes in number of words or changes in duration of utterances over time. Second, Kruskal Wallis rank sum test was performed in order to examine iconic gesture rates over time. We calculated gesture rates as the number of gestures per 100 words. Finally, descriptive statistics were used to examine how iconicity types were distributed across timepoints. Iconicity types were calculated as percentages (iconicity count/total number of iconic gestures * 100). For instance, if a participant produced a total of 4 gestures three months post injury, with one enacting and two handling, and one mixed (using 2 hands simultaneously), this equated to 25% enacting, 50% handling, and 25% mixed. We chose to calculate percentages to examine how iconicity types are distributed, which reveals further information about how iconic gestures are being utilized. Higher percentages of handling gestures may suggest that conveying tool-based concepts via gestures are prioritized more than other iconicity types (e.g. gestures that denote path or shape). Gesture rates and the calculation for descriptive statistics were repeated for the subsequent aims. Supplemental gestures were included in the descriptive statistics as percents. For instance, if 10 gestures were produced at a given timepoint and only 2 were supplemental, the total percentage is reported as 20%. This indicates that a majority of the gestures were redundant to speech.

#### Aim 2: identify the extent to which language impairment relates to iconic gesturing in TBI

We hypothesized that participants with more severe language impairment would produce higher iconic and supplemental gesture rates. Neuropsychological assessments were only assessed at three months post injury. Thus, Spearman correlations for n = 31 individuals were conducted to examine the relationship between neuropsychological assessments and two gesture variables (iconic and supplemental gesture rates). Due to the categorical nature of iconicity types, and their infrequency, correlations between iconicity types and neuropsychological data were not conducted. Correction for multiple comparison was not performed.

#### Aim 3: characterize the extent to which iconic gesturing changes across TBI recovery

We hypothesized that in a paired longitudinal analysis, gestures will not significantly change across a two-year recovery, from sub-acute (three months) to chronic (two years). This was evaluated in Group 1 (n = 14), and Group 2 (n = 6). Due to varying of n values for each timepoint, we selected the two time points that retained the most individuals. This was timepoint three and 24-months (Group 1; n = 14). Demographic and neuropsychological data for the fourteen individuals in this sample is seen in Table 3. In this sample, a paired Wilcoxon signed rank test was performed to examine differences in iconic and supplemental gestures at three months and two years post injury. Additionally, descriptive statistics examined the use of iconicity types at three months and 24-months post injury. Because of their tendency to produce few items per category, we did not further perform statistical analyses to determine significant differences in iconicity across the two timepoints. As a highly exploratory second subgroup, Group 2 comprised individuals who had data at every timepoint (n = 6). Demographic and neuropsychological data for the six individuals in this sample are seen in Table 4. In this sample, a Kruskal-Wallis rank sum test was performed to determine any differences in iconic gesture rates between any of the timepoints. Additionally, descriptive statistics examined iconicity types produced at each timepoint, as described earlier in Group 1.

**TABLE 3:** Demographic and neuropsychological data for Group 1 (*n* = 14).

Factor	M(SD) or N (%)
**Post Traumatic Amnesia (Days)**	
Mean (SD)	46.1 (20.5)
Min, Max	14.0, 90.0
**Sex**	
Female	2 (14.3%)
Male	12 (85.7%)
**Age**	
Mean (SD)	33.5 (14.5)
Median [Min, Max]	27.5 [17.0,59.0]
**Head Injury**	
Closed	13(92.9%)
Open and closed (multiple injuries)	1 (1%)
**Language**	
Monolingual	11 (78.6%)
Bilingual	2 (14.3%)
Multilingual	1 (7.1%)
**Western Aphasia Battery—Aphasia Quotient**	***Max Score* = *100 (higher* = *milder aphasia)***
Mean (SD)	92.9 (3.35)
Median [Min, Max]	93.8 [84.0, 97.0]
Missing	1 (7.1%)
**Boston Naming Test**	***Max Score* = *60 (higher* = *milder anomia)***
Mean (SD)	39.8 (13.2)
Median [Min, Max]	42.0 [10.0, 58.0]
Missing	1 (7.1%)
**Verb Naming Test**	***Max Score* = *22 (higher* = *milder anomia)***
Mean (SD)	20.3 (2.59)
Median [Min, Max]	21.0 [21.0, 22.0]
Missing	1 (7.1%)
**LaTrobe Communication Questionnaire—Self**	***Max Score* = *120 (higher* = *more perceived issues)***
Mean (SD)	49.0 (12.8)
Median [Min, Max]	46.0 [33.0, 72.0]
Missing	1 (7.1%)
**Dysarthria**	
Present	12 (85.7%)
Not Present	1 (7.1%)
Missing	1 (7.1%)
**Apraxia of speech**	
Present	0
Not Present	12 (85.7%)
Missing	2 (14.3%)
**Initial Glasgow Comma Scale**	***Max* = *15 (higher score* = *higher functioning)***
Mean (SD)	6.73 (3.47)
Median [Min, Max]	6.50 [3.00, 14.0]

**TABLE 4:** Demographic and neuropsychological information for Group 2 (*n* = 6).

Factor	M(SD) or N (%)
**Post Traumatic Amnesia Days**	
Mean (SD)	54 (28.2)
Min, Max	14.0, 90.0
**Sex**	
Female	1 (16.6%)
Male	5 (83.3%)
**Age**	
Mean (SD)	40.5 (12.5)
Min, Max	23,56
**Head Injury**	
Closed	6 (100%)
**Language**	
Monolingual	5 (83.3%)
Bilingual	1 (16.6%)
**Western Aphasia Battery—Aphasia Quotient**	***Max Score* = *100 (higher* = *milder aphasia)***
Mean (SD)	92.0 (4.64)
Median [Min, Max]	93.8 [84.0, 97.0]
**Boston Naming Test**	***Max Score* = *60 (higher* = *milder anomia)***
Mean (SD)	43.6 (17.0)
Median [Min, Max]	93.8 [10.0, 58.0]
**Verb Naming Test**	***Max Score* = *22 (higher* = *milder anomia)***
Mean (SD)	19.6 (3.3)
Median [Min, Max]	21.0 [13, 21]
**LaTrobe Communication Questionnaire—Self**	***Max Score* = *120 (higher* = *more perceived issues)***
Mean (SD)	44.1 (7.68)
Median [Min, Max]	46.0 [37, 56]
**Dysarthria**	
Present	6 (100%)
**Apraxia of speech**	
Not Present	5 (83.3%)
Missing	1 (16.7%)
**Initial Glasgow Comma Scale**	***Max* = *15 (higher score* = *higher functioning)***
Mean (SD)	6.6 (4.32)
Median [Min, Max]	5.50 [3.00, 14.0]

#### Case study

Notably, there was a single individual that scored in the Western Aphasia Battery-Revised as having moderate-severe aphasia and appeared to have more challenges in word finding compared to the rest of the participants. Thus, we reported on gesture production on this individual to examine gesture use more readily during narrative production in a person with more impaired language.

## Results

### Number of words and duration produced over time

Kruskal-Wallis rank sum test was performed in order to identify differences in number of words, duration of utterances (in seconds) and, gesture rates (number of gestures per 100 words). For the larger N = 54 group, there were no significant differences in the number of words produced χ^2^(4, *N* = 54) = 8.0, *p* = 0.09 or the duration of utterances across timepoints χ^2^(4, *N* = 54) = 6.69, *p* = 0.15. See Table 5 for the descriptive statistics. For the paired n = 14 group, there were also no significant differences in the number of words used across timepoints (V = 52.5, p > 0.99) or the duration of utterances across timepoints (V = 56, p = 0.48). For the paired n = 6 group, there was significant difference in the number of words produced across timepoints χ^2^(4, *N* = 6) = 9.43, *p* = 0.05. Lastly, there were no significant differences in the duration of utterances χ^2^(4, *N* = 6) = 8.61, *p* = 0.07.

**TABLE 5:** Descriptive statistics for number of words, duration of utterances, and gesture rates across timepoints.

Gesture Type	Timepoint				
	**Three** **Months** ***n* = 31**	**Six Months** ***n* = 31**	**Nine Months** ***n* = 16**	**Twelve Months** ***n* = 36**	**Twenty-Four Months** ***n* = 30**
Number of words	M = 68.58 ± 48.7 Med = 46.0 Min = 17.0 Max = 212	M = 103.16 ± 193.3 Med = 65.0 Min = 27.0 Max = 1127	M = 428 ± 1134 Med = 10.0 Min = 0.00 Max = 4043	M = 59.3 ± 32.4 Med = 57.0 Min = 9.0 Max = 141	M = 60.5 ± 31.08 Med = 60.0 Min = 12 Max = 134
Duration of utterances (seconds)	M = 25.84 ± 15.45 Med = 24.0 Min = 5.00 Max = 59.0	M = 34.19 ± 47.58 Med = 22.0 Min = 9.0 Max = 279.0	M = 142 ± 369 Med = 6.0 Min = 0.00 Max = 1321	M = 21.7 ± 14.31 Med = 19 Min = 0.00 Max = 50.0	M = 21.4 ± 15.7 Med = 18.5 Min = 3.0 Max = 66.0
Iconic gesture rates	M = 4.98 ± 4.81 Med = 4.76 Min = 0 Max = 16.67	M = 4.02 ± 4.64 Med = 1.69 Min = 0 Max = 16.13	M = 1.50 ± 3.70 Med = 0 Min = 0 Max = 11.63	M = 4.98 ± 6.92 Med = 4.06 Min = 0 Max = 36.36	M = 5.56 ± 7.29 Med = 3.73 Min = 0.00 Max = 30.8

### Aim 1: characterize the extent to which adults with moderate-severe TBI produce iconic gestures

**FIGURE 3:**
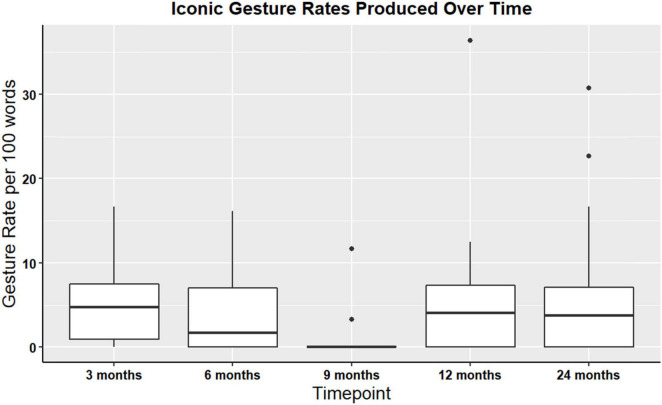
Boxplot showing overall iconic gesture rates for participants with gesture data at all timepoints TBI (non-paired sample; *N* = 54).

Iconic and supplemental gesture rates were assessed at all timepoints among the n = 54 participants (non-paired dataset). Iconic and supplemental gestures were present across all time points. There were no significant differences in iconic gesture rates over time χ^2^(4, *N* = 51) = 5.89, *p* = 0.21, or in supplemental gesture rates over time χ^2^(4, *N* = 54) = 4.1, *p* = 0.39. For a visual representation of iconic gesture rates over time, see Figure 3. Additionally, descriptive statistics were calculated for the iconicity types: enacting, handling, object, shape, path, and mixed. Enacting and handling were the most frequently produced, and this pattern was consistent at each timepoint. Table 6 denotes descriptive statistics for supplemental gestures, and iconicity types.

**TABLE 6:** Descriptive statistics for supplemental and iconicity types at all timepoints.

Gesture Type	Timepoint				
	**Three** **Months** ***n* = 31**	**Six Months** ***n* = 31**	**Nine Months** ***n* = 16**	**Twelve Months** ***n* = 36**	**Twenty-Four Months** ***n* = 30**
Supplemental (% Total)	M = 7.5% ± 22.4% Med = 0%	M = 2.11% ± 8.14% Med = 0%	M = 5.38% ± 14.5% Med = 0%	M = 5.24% ± 9.68% Med = 0%	M = 6.88% ± 22.75% Med = 0%
Enacting (%)	M = 69.0%0.4 ± 28.5% Med = 66.6%	M = 55.9% ± 33.30% Med = 55.1%	M = 55.7% ± 34.4% Med = 63.3	M = 67.0% ± 29.8% Med = 66.6%	M = 61.7% ± 28.3% Med = 56.3%
Handling (%)	M = 12.8% ± 16.8% Med = 0%	M = 16.0% ± 25.48% Med = 0%	M = 25.4% ± 32.7% Med = 8.33%	M = 24.09% ± 26.1% Med = 16.7%	M = 17.9% ± 26.7% Med = 0%
Object (%)	M = 8.27% ± 13.4% Med = 0%	M = 16.6% ± 16.4% Med = 15.34%	M = 4.04% ± 8.91% Med = 0%	M = 3.33% ± 8.82% Med = 0%	M = 11.6% ± 12.7% Med = 7.14%
Shape (%)	M = 0.57% ± 2.66 Med = 0%	Not produced	M = 8.93% ± 27.0% Med = 0%	M = 1.10% ± 5.46% Med = 0%	M = 1.25% ± 5.59% Med = 0%
Path (%)	Not produced	M = 0.41% ± 1.94% Med = 0%	Not produced	M = 1.59% ± 7.27% Med = 0%	M = 3.5% ± 8.60 Med = 0%
Mixed (%)	M = 8.90% ± 23.9% Med = 0%	M = 11.1% ± 21.5% Med = 0%	M = 5.48% ± 16.3% Med = 0%	M = 2.83% ± 7.29% Med = 0%	M = 4.08% ± 8.49% Med = 0%

### Aim 2: identify the extent to which language impairment relates to iconic gesturing in TBI

Spearman’s rank correlations were computed to examine the relationship between gesture variables at three months post injury and neuropsychological assessment scores. Spearman correlation indicated a negative correlation between the BNT and iconic gesture rates (see Table 7). No other significant correlations were noted.

**TABLE 7:** Spearman correlations between neuropsychological assessment scores and gestures at 3 months post TBI (*n* = 31).

Gesture Types	Western Aphasia Battery- Aphasia Quotient	Boston Naming Test	Verb Naming Test	La Trobe Communication Questionnaire-Self
Supplemental gesture rate	r_s_ = −0.05, *p* = 0.61	r_s_ = −0.03, *p* = 0.76	r_s_ = 0.09, *p* = 0.35	r_s_ = 0.02, *p* = 0.84
Iconic gesture rate	r_s_ = −0.03, *p* = 0.77	r_s_ = −0.20, *p* = 0.03	r_s_ = 0.002, *p* = 0.97	r_s_ = 0.02, *p* = 0.84

### Aim 3: characterize the extent to which iconic gesturing changes across TBI recovery

#### Group 1: *n* = 14

**FIGURE 4:**
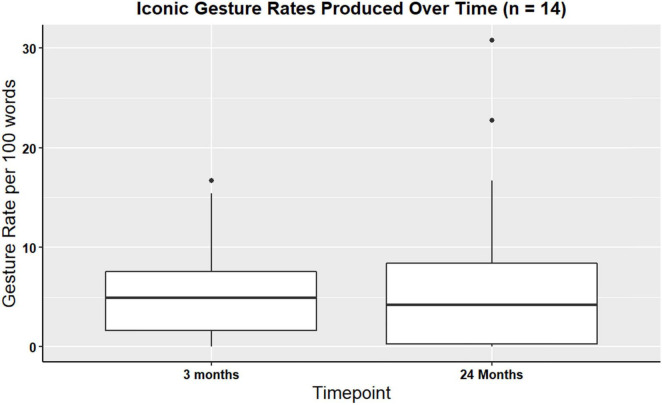
Boxplot showing overall iconic gesture rates for participants with gesture data at three- and 24-months post TBI (*n* = 14).

Wilcoxon signed rank test was computed to identify longitudinal changes in iconic and supplemental gesture use. Wilcox signed rank test did not identify changes in iconic (V = 39, p > 0.99) or supplemental (V = 4, p = 0.86) gesture use over time. That is, the production of iconic gestures during the procedural narrative across a two-year period of recovery did not significantly change. For a visual representation of iconic gesture rates see Figure 4.

Descriptive statistics were calculated to examine iconicity types produced across both timepoints. Consistent with previous findings, enacting, handling, and object gestures were more frequently produced compared to other types at timepoints three and 24 months. Note that no statistical analyses were performed. Table 8 shows descriptive statistics for iconicity use at timepoints three and 24 months.

**TABLE 8:** Descriptive statistics of iconicity types in Group 1 (*n* = 14) between timepoints three and 24-months.

Iconicity types (%)	Three Months	Twenty-Four Months
Enacting	M = 75.3% ± 24.98% Med = 66.7%	M = 58.4% ± 29.9% Med = 56.25%
Handling	M = 11.5% ± 16.6% Med = 0%	M = 20.25% ± 33.6% Med = 0%
Object	M = 7.95% ± 16.1% Med = 0%	M = 12.3% ± 13.5% Med = 10%
Shape	M = 1.14% ± 3.77% Med = 0%	Not produced
Path	Not produced	M = 4.5% ± 9.56% Med = 0%
Mixed	M = 4.17% ± 10.4% Med = 0%	M = 4.5% ± 9.56% Med = 0%

#### Group 2: *n* = 6

**FIGURE 5:**
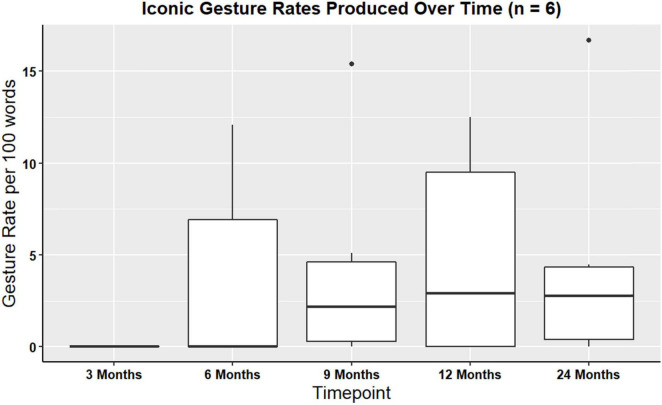
Boxplot showing overall iconic gesture rates produced for participants with gesture data at all timepoints (*n* = 6).

A Kruskal-Wallis rank sum test was performed in order to examine iconic and supplemental gesture rates over time. There were no significant differences in iconic gesture rates over time χ^2^(4, *N* = 6) = 2.13, *p* = 0.71, or in supplemental gesture rates over time (χ^2^(4, *N* = 6) = 1.29, *p* = 0.86). Figure 5 shows visual representations of iconic gesture rates.

Descriptive statistics were calculated to determine iconicity types produced at all timepoints. Like previous findings, enacting, handling, and object were the iconicity types that were produced often compared to the other iconicity types. This trend was consistent across a two-year period. Table 9 shows descriptive statistics (e.g., mean, median, standard deviation) for iconicity types produced at all the timepoints.

**TABLE 9:** Descriptive statistics of iconicity types (*n* = 6) between all timepoints.

Iconicity Types (%)	Timepoint				
	**Three** **Months**	**Six Months**	**Nine Months**	**Twelve Months**	**Twenty-Four Months**
Enacting	M = 70.8% ± 21.0% Med = 66.7%	M = 39.8% ± 20.9% Med = 39.8%	M = 16.67% ± 23.6% Med = 16.7%	M = 27.8% ± 25.5%% Med = 33.3%	M = 72.5% ± 32.0%% Med = 75%
Handling	M = 8.33% ± 16.7% Med = 0%	M = 4.55% ± 6.43% Med = 4.55%	M = 83.33% ± 23.6% Med = 83.3%	M = 44.4% ± 26.8% Med = 33.3%	M = 22.5% + 26.3% Med = 20%
Object	M = 12.5% ± 25% Med = 0%	M = 19.9% ± 10.4% Med = 19.9%	Not produced	M = 11.1% ± 19.2% Med = 0%	M = 5.00% + 10.0% Med = 0%
Shape	Not produced	Not produced	Not produced	M = 8.33% ± 14.4% Med = 0%	Not produced
Path	Not produced	M = 4.55% ± 6.43% Med = 4.55%	Not produced	Not produced	Not produced
Mixed	M = 8.33% ± 16.7% Med = 0%	M = 31.3% ± 44.2% Med = 31.3%	Not produced	M = 8.33% ± 14.4% Med = 0%	Not produced

### Case study

A single participant (21-year-old male) used more supplemental gestures than the rest of the group. This individual had the lowest aphasia quotient score from the WAB-R, which was an Aphasia Quotient score of 55, indicating moderate-severe aphasia (the cut-off for “severe” is a score of 50). This participant also scored as the most severe of the group on the Boston Naming Test and Verb Naming Test (scores of 0/30, and 3/22, respectively), indicating severe anomia. At three months most injury, 100% of the gestures this participant produced were supplemental to speech. At six months, 37.5% of the gestures elicited were supplemental to speech, and at nine months, 50% of the gestures produced were supplemental to speech. Compare this with the entire group (in Aim 1, N = 54), where the average was 7.5% (SD = 22.4%) at three months, 2.11% (SD = 8.14%) at six months, and 5.38% (SD = 14.5%) at nine months.

At three months post injury, a total of five iconic gestures were produced, of which 40% were enacting, 40% were handling, and 20% were object. At six months, a total of five iconic gestures were produced, of which 62.5% were enacting, 12.5% were handling, 25% were object. At nine months, a total of six iconic gestures were produced, of which 50% were enacting, 16.6% were handling, 16.6% were object, and 16.6% were a mixture of iconicity types. These results show a trend of increase in enacting gestures (+ 10% after three months) and mixed iconicity types (+ 16.6% after two months). Additionally, there was a decrease in handling (−24%) and decrease in object (−4%). Path and shape iconicity were not elicited by this participant.

## Discussion

### Aim 1: characterize the extent to which adults with moderate-severe TBI produce iconic gestures

We characterized iconic gesture rates and iconicity types in adults with moderate-severe TBI, having no directional hypothesis. Participants utilized iconic gestures and used a variety of iconicity types. Participants used enacting and handling gestures the most often, which are iconicity types that are semantically most related to the task. This finding was consistent in our larger (N = 54) and our smaller (*n* = 14 and *n* = 6) samples. Interestingly, iconic gesture rates reported in our study resembled the rates that were reported in previous literature on gesture in post stroke aphasia (Stark and Cofoid, 2022). This similarity suggests that individuals with TBI may produce gestures similarly to those with stroke-aphasia. However, it is important to note that in the Stark and Cofoid study, the mean years post injury was five, and the mean years of speech language pathology treatment was three. Thus, future research comparing etiologies is necessary, which will aid in developing therapeutic interventions and rehabilitation strategies that are tailored for TBI and stroke populations.

Iconicity types provide meaningful information during the task and showed surprising between-participant similarity during the task. For instance, participants would pretend to close a sandwich by either closing both hands together or flipping one hand over. These gestures were characterized as enacting. A common example of mixed iconicity use across the sample was using one hand to represent scooping vegemite out of the jar (enacting), pretending to use a knife to spread the vegemite on a piece of bread (handling), while pretending to hold bread in the other hand (object). Some speakers used their palm and gestured a circular motion to demonstrate how they would spread the vegemite on the bread. These gestures enriched overall communication by incorporating visual cues that complemented the spoken descriptions, though typically in a redundant and not supplemental fashion. These findings support the idea that gesture and speech are highly integrated processes in moderate-to-severe TBI, and that iconic gestures occur ubiquitously with speech just as has been shown in cognitively healthy adult populations.

In a recent study on gesture and speech integration, adults with moderate-severe TBI were shown stories with accompanying gestures, and then asked to retell the stories immediately after and after a short delay (Clough et al., 2023). In this study, it was noted that individuals with TBI were as likely to employ gestures when retelling stories as their healthy peers. While our study focuses on spontaneous gesture production during a procedural narrative, rather than retellings of stories produced by a narrator who gestured (as was the case in the Clough study), our findings complement Clough et al., 2023, in that they support that the capacity and meaningfulness of gesture to enhance speech can be largely intact in persons with moderate-to-severe TBI.

### Aim 2: identify the extent to which language impairment relates to iconic gesturing in TBI

We hypothesized that participants with more severe language impairment would produce more iconic and supplemental gestures. When evaluating this in the sample at three months (n = 31), we did not find significant correlations between gestures (i.e., iconic and supplemental) and most of the neuropsychological assessments examining language and communication, with the exception of the Boston Naming Test. These findings were dissimilar to findings from a prior study, which found negative correlations between scores on the Test of Adolescent/Adult Word Finding and total occurrence of all gestures (Kim et al., 2015). They suggested that this indicated that poorer word finding post-TBI resulted in higher gesture rates. This finding was consistent with findings from post-stroke aphasia, where individuals with lower scores on word finding assessments have been shown to employ more gestures than their healthy peers (e.g., Sekine et al., 2013).

Note that gestures were annotated during a picture naming task in the Kim et al., 2015, study, which is a confrontation task requiring individuals with TBI to name the picture in front of them. This type of task constrains the speaker immensely, in that there is a correct target (i.e., if shown a picture of a lion, you must say ‘lion’ to get full points). In procedural narratives, individuals can ‘talk around’ (circumlocute), or avoid saying a word that is difficult for them to find. As such, they may demonstrate far fewer instances of gesturing because they are providing words that are easier to access or have sufficient flexibility in describing something using other words that still convey the point. For example, a person might say, “You get that… you know, the thing made from peanuts in a jar, and you spread it on the bread,” thus conveying the target of “peanut butter” without having to utter that specific two-word phrase.

Our findings contrast prior TBI and aphasia research, as overall, we did not find significant strong correlations between gesture use and most language assessment scores. Indeed, participants appeared to largely produce gestures redundant with speech, as is more typical of adults without aphasia (Kong et al., 2015). However, of note is that only 21 of 54 (38.8%) had clinical aphasia by the Western Aphasia Battery-Revised standard, indicating that the majority of the TBI sample had few, if any, language impairments. The percentage of persons with aphasia in the current moderate-to-severe TBI sample is not atypical, with estimates widely ranging on the number of persons with TBI experiencing aphasia, largely due to the heterogeneity of TBI etiology, location (e.g., left or right hemisphere), and severity. A recent retrospective analysis among US service personnel with complicated mild-to-severe TBI found that 1.5% demonstrated aphasia (Lindsey et al., 2023) whereas another, larger study of veterans found that, of those diagnosed with a TBI of varying etiologies and severities, up to 77% also presented with aphasia (Norman et al., 2013). Notably, the case study individual with most severe language impairment in our TBI group presented with higher supplemental gesture rates than the rest of the group, suggesting that in the case of moderate-severe language impairment in TBI, gesture may be utilized to supplement speech and resemble the post-stroke aphasia pattern. This participant produced a variety of iconicity types at three-, six-, and nine- months post injury, and did so consistently across time. This participant used mostly enacting, handling, and object gestures, which are iconicity types that are semantically most related to the task, indicating that gesture accompanied speech in a meaningful manner. For instance, this participant would use his hand to pretend to interact with the bread and say, “you get the thing, I don’t know what you call it”, while eliciting an enacting or handling gesture. This indicates that in this participant’s case, gestures seemed to aid in conveying information that could not be conveyed via speech. Findings from this participant are consistent with previous TBI and post-stroke aphasia research, showing that when language is severely impacted, no matter the etiology (i.e., TBI or stroke), gesture becomes an important tool for conveying information that is not available in speech.

### Aim 3: characterize the extent to which iconic gesturing changes across TBI recovery

The hypothesis driving this aim was that gestures would not significantly change over time and was evaluated in two subgroups of persons (*n* = 14, comparing three- and 24-months post-TBI; and *n* = 6, comparing all timepoints post-TBI). As predicted, there were no longitudinal changes in iconic gestures or supplemental gestures. Another way of understanding this would be to say that individuals with TBI have the capability of producing iconic gestures which can, if need be, supplement speech, as early as three months post-TBI, and that this ability remains stable across recovery.

This is a highly exploratory finding in a very small sample and should be interpreted accordingly, and ideally replicated in a much larger sample.

Existing literature has indicated that in cases of moderate-severe TBI, there are often poorer cognitive and psychosocial outcomes following one year after brain injury (Dahlberg et al., 2006; Rapoport et al., 2006). However, the present study suggests that multimodal communication may be relatively resilient post-TBI, unlike some cognitive and linguistic functions. This is a particularly encouraging finding that suggests the importance of assessing multimodal communication post-TBI. Indeed, other researchers and clinicians have noted that multimodal communication is imperative to evaluate after brain injury, developing assessments that more readily characterize gesture produced spontaneously during tasks (Caute et al., 2021; Hogrefe et al., 2021), given that most standardized batteries of language and communication neglect gesture. Further, given the relationship of gesture for improving language, memory, and learning, as described in the Introduction, this exploratory finding suggests that rehabilitation professionals (e.g., speech-language therapists) might leverage intact multimodal communication skills to improve other outcomes, such as cognitive and verbal communication impairments.

### Elaborating on gesture and speech models

In a recent evaluation of the AR-Sketch Model (de Ruiter, 2017), which postulates that iconic gestures are assumed to express information that is redundant to speech in cognitively healthy speakers, it was found that persons with aphasia did not conform (de Beer et al., 2020). Instead, persons with aphasia tended to use iconic gestures in a compensatory manner for speech (i.e., to supplement speech), leading de Beer and authors to argue that aphasia fundamentally changes the relationship of gesture and speech. The evidence provided in our study of persons with TBI, of which fewer than half had aphasia, provides some evidence to support both of these ideas. Firstly, it was found that most individuals with TBI, even those with clinical aphasia, tended to use iconic gestures in a redundant manner. This coincides with the assumptions of the AR-Sketch Model. One such reason for this may have been that most did not have clinical aphasia, and for those who did, they presented with relatively mild aphasia. In this study, the mean Aphasia Quotient from the Western Aphasia Battery-Revised was 92.5 ± 7.4, suggesting the presence of mild aphasia (the cut-off to consider one to have clinical aphasia is a score of less than 93.8). The wide range of scores (55 – 100) suggest that a few individuals had more severe aphasia, but the small standard deviation (7.4) suggests that most experienced relatively mild aphasia. This then leads us to a second conclusion, broadly in support of de Beer et al. (2017), who stipulate that aphasia changes the relationship of speech and gesture. The case study presented here, of the individual with moderate-severe aphasia (score of 55) after TBI, demonstrated production of a high amount of supplemental iconic gestures. As such, the evidence provided by the current study extends de Beer and colleagues’ assumption by narrowing the focus slightly: a critical ‘tipping point’ must exist, such that there is some *level* of aphasia severity that changes the nature of speech and gesture. Future work is needed to identify where that ‘tipping point’ of aphasia severity lies.

## Limitations and future directions

A limitation of this study is the absence of a control group, which would have allowed for a direct comparison of gesture use during the task between speakers with and without TBI. Another limitation of this study is its exclusive focus on a single task, specifically procedural narratives. It is probable that the utilization of gestures, especially representational gestures, may differ across various tasks (Stark and Cofoid, 2022), suggesting the need for further research to examine task variation and its impact on gesturing in post-TBI. An additional limitation of this study is that it relies on retrospective data analysis, with many videos being unusable due to poor visibility of the participants’ hands. This highlights the urgent need for studies to consider hand gestures when collecting video data. There is a clear need for more high-quality video data in TBI research to ensure better visibility and accuracy in gesture analysis.

Future research should investigate longitudinal changes in larger sample sizes, empirically investigating gesture use across different brain injury etiologies and across a variety of tasks with varying difficulties (Stark et al., 2021). For instance, future research could evaluate the extent to which iconic gestures increase when the *task* presented is challenging, such as a task requiring an attention split. In the case of severe language impairment, it could be posited that gesture production increases due to the higher cognitive demands associated with language production. If gestures are indeed generated in response to increased cognitive demands across various tasks, this would serve as additional evidence that gestures not only complement speech, but also can be employed to alleviate cognitive burden during moments of increased load.

## Conclusion

This study demonstrates that individuals with moderate-to-severe TBI utilize iconic gestures during spontaneous speech, and those that tend to use supplemental gestures may also be those with more impaired language processes. Exploratory and highly preliminary evidence from two small samples suggests that iconic gesture use may not change across a recovery period of 2 years.

## Data Availability

The datasets presented in this study can be found in online repositories. The names of the repository/repositories and accession number(s) are: https://osf.io/58gqx/. The raw data was obtained from tbibank: https://tbi.talkbank.org/.
